# *De novo* assembly and characterization of the complete chloroplast genome of *Elymus magellanicus* (É.Desv.) Á.Löve, 1984 (Poaceae, Pooideae)

**DOI:** 10.1080/23802359.2022.2135400

**Published:** 2022-10-27

**Authors:** Xiaojun Wu, Xiangdong Chen, Zhongwen Huang, Cuicui Ren, Tiezhu Hu, Zhengang Ru

**Affiliations:** a Center of Wheat Research, Henan Institute of Science and Technology, Xinxiang, China; b Henan Key Laboratory of Hybrid Wheat, Xinxiang, China; c Henan Collaborative Innovation Center of Modern Biological Breeding, Xinxiang, China

**Keywords:** *Triticeae*, chloroplast genome, grass, phylogenetic analysis

## Abstract

*Elymus magellanicus* (É.Desv.) Á.Löve is a foliage accent plant that originated in South America. In this study, the complete chloroplast genome of *E. magellanicus* is reported. It was found to have a total size of 133,249 bp. The chloroplast genome was found to consist of two inverted repeats (IRA and IRB) of 21,421 bp each, a small single-copy region of 12,709 bp, and a large single-copy region (77,698 bp). The annotation results show the GC content of the chloroplast genome to be 38.47%, including 40 tRNA genes, 82 protein-coding genes, and 8 rRNA genes. Phylogenetic analysis of 29 species revealed that *E. magellanicus* is closely related to *E. arenarius*.

*Elymus magellanicus* (É.Desv.) Á.Löve is a perennial grassy herb that is mainly distributed in South America. Because of its distinctive metallic-blue leaves, it is commonly used as a foliage plant in China. *E. magellanicus* is an allotetraploid with an SSHH genome type (Jensen 1993) that has many excellent traits such as drought tolerance, salt-alkali tolerance, barren tolerance, and disease resistance. In order to introduce its excellent disease resistance and adaptability characteristics into crops, some wheat researchers have tried to cross *E. magellanicus* with wheat in China. Thus far, there have been no reports of successful distant hybridization. However, the conservative nature of the chloroplast genome has made it a valuable source for taxonomic classification and phylogenetic studies of plant species (Clegg et al. 1994; Zhang et al. 2016). In this study, the chloroplast genome of *E. magellanicus* was sequenced, assembled, and annotated to identify its phylogenetic position among the 29 selected species. We hope to provide some useful references for its distant hybridization utilization.

In this study, fresh leaves of *E. magellanicus* were collected from the Botanical Garden of Center of the Wheat Research, Xinxiang, Henan, China (113°88′E 35°30′N). The voucher specimen was deposited at the Herbarium of Henan Institute of Science and Technology, Xinxiang, China (Zhengang Ru; rzgh5819@163.com) under the voucher number XM-W046. The total genomic DNA of the fresh leaves was extracted according to a modified CTAB method (Doyle and Doyle 1987). The constructed whole-genome DNA sequencing library was sequenced using the Illumina NovaSeq 6000 platform at Novogene (Beijing, China). These efforts produced 11.31 Gb of clean data. The chloroplast genome of *E. magellanicus* was assembled using the NovoPlasty v.4.3.1 software package (Dierckxsens et al. 2017) and annotated with CpGAVAS2 (Shi et al. 2019). The sequence data and gene annotations were submitted to the GenBank database under accession number MZ337548.

The complete chloroplast genome of *E. magellanicus* was a 133,249-bp circular molecule with 38.47% GC content, containing two inverted repeats (IRA and IRB) of 21,421 bp each separated by a small single-copy region (12,709 bp) and one large single-copy region (77,698 bp). The chloroplast genome encoded a set of 130 functional genes, including 40 tRNA genes, 82 protein-coding genes, and 8 rRNA genes. Among them, 21 genes present in IRs were found to be duplicated (eight tRNA genes, nine protein-coding genes, and four rRNA genes).

To assess the phylogenetic relationship of *E magellanicus*, a total of 29 chloroplast genomes of Poaceae were aligned using Clustal Omega software (Sievers and Higgins 2014), and the maximum likelihood method was used for phylogenetic analysis with 1000 bootstrap replicates using PhyML 3.0 software (http://www.atgc-montpellier.fr/phyml/) (Guindon et al. 2010). The results showed that *E. magellanicus* was most closely related to *E. arenarius*, and it formed a single clade with *E. arenarius* and *E. chinensis* (Figure 1). The phylogenetic tree might provide a reference for the selection of bridging species in distant hybridization between *E. magellanicus* and wheat.

**Figure 1.**
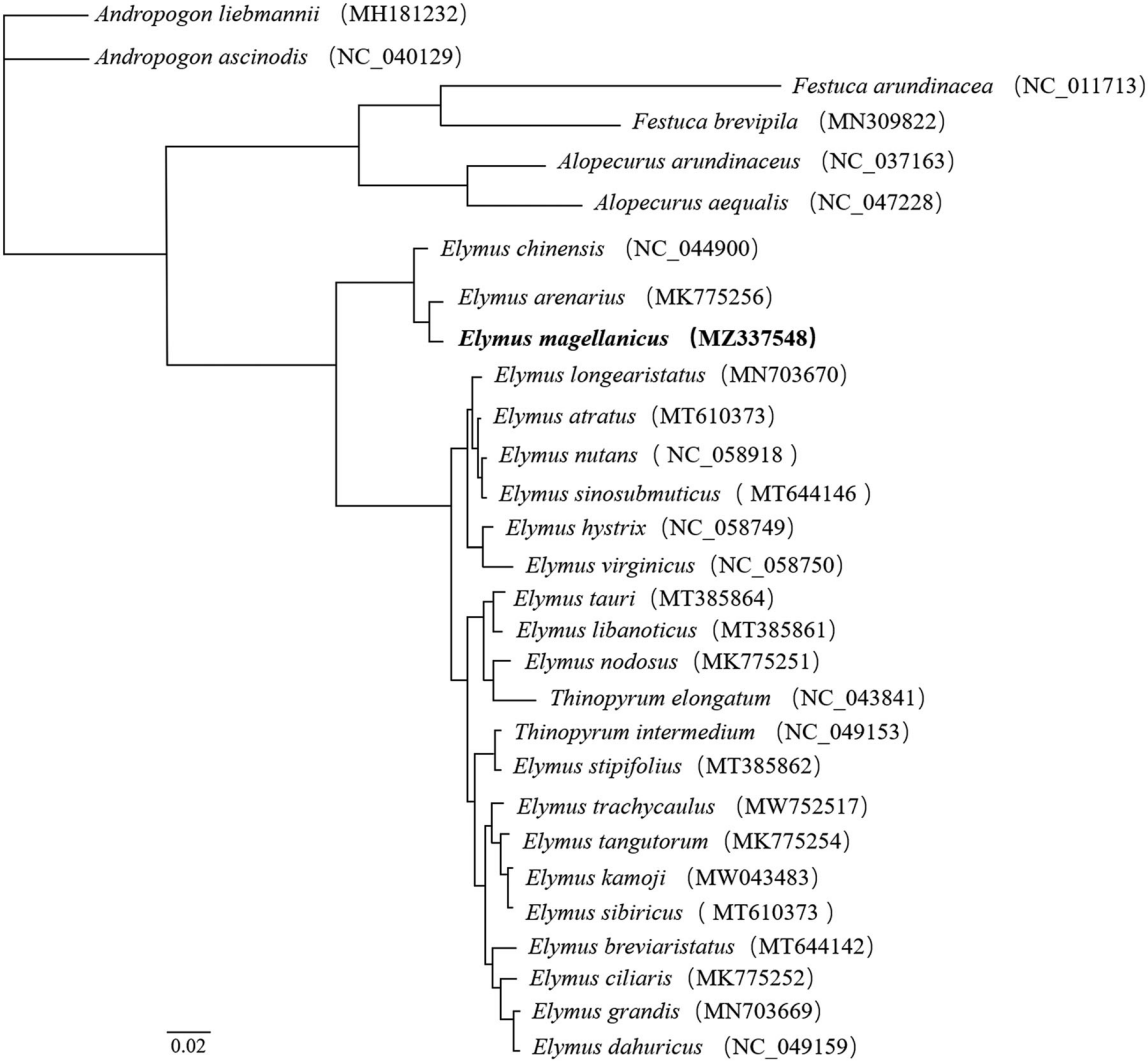
The maximum likelihood (ML) phylogenetic tree was constructed by the complete chloroplast genome of 29 species, including the *Elymus magellanicus* (MZ337548) chloroplast genome in this study.

## Data Availability

The genome sequence data that support the findings of this study are openly available in GenBank of NCBI at https://www.ncbi.nlm.nih.gov/, reference number MZ337548. The associated BioProject, Bio-Sample, and SRA numbers are PRJNA783482, SAMN23441549, and SRP350763, respectively.
